# P-339. Infection Control Link staff program that involves all professionals improves the quality of infection control in hospitals

**DOI:** 10.1093/ofid/ofae631.541

**Published:** 2025-01-29

**Authors:** Michinori Shirano, Hidenori Nakagawa, Ko IIida, Tomohiro Asaoka, Risa Fukuoka, Ryo Morita, Naoki Yamaguchi, Sumiyo Nanri, Mika Imasaki, Naomi Yamaguchi, Tetsushi Goto

**Affiliations:** Osaka City General Hospital, Osaka, Osaka, Japan; Osaka City General Hospital, Osaka, Osaka, Japan; Osaka City General Hospital, Osaka, Osaka, Japan; Osaka City General Hospital, Osaka, Osaka, Japan; Osaka City General Hospital, Osaka, Osaka, Japan; Osaka City General Hospital, Osaka, Osaka, Japan; Osaka City General Hospital, Osaka, Osaka, Japan; Osaka City General Hospital, Osaka, Osaka, Japan; Osaka City General Hospital, Osaka, Osaka, Japan; Osaka City General Hospital, Osaka, Osaka, Japan; Osaka City General Hospital, Osaka, Osaka, Japan

## Abstract

**Background:**

Collaboration among all medical staff is essential to improve the quality of infection control. However, it is not easy to fully disseminate infection control knowledge to all. The purpose of this study was to evaluate whether the implementation of the Infection Control Link staff program organized in our hospital improved the quality indicator (QI) of infection control.

**Figure:**
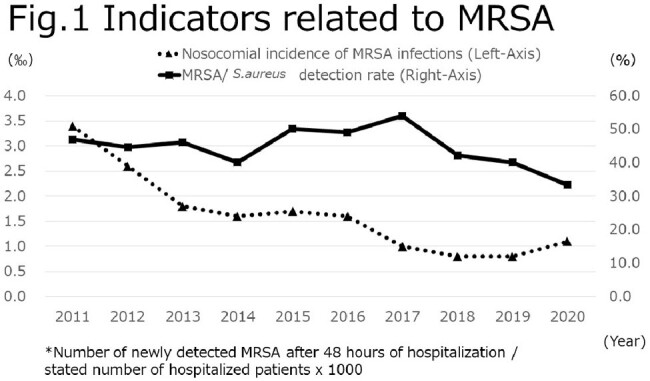

**Methods:**

Our hospital's link staff program is unique in that it is composed of all healthcare professions, including not only physicians and nurses, but also laboratory technicians, pharmacists, radiologists, and physical therapists. The program was launched in 2012. Various lectures and hands-on learning experiences (hand hygiene compliance audit experience, personal protective equipment (PPE) donning and doffing training, etc.) were conducted at monthly meetings, and each department was asked to take the training home and put it into practice. As QI for infection control included the number of new hospital-acquired cases of Methicillin-resistant *Staphylococcus aureus* (MRSA) infection, the percentage of MRSA among all *S. aureus* detected in culture, the number of blood culture tests per 1000 patients/day, two sets of blood culture collection rate were used as indicators to evaluate efforts; QI data through 2020 were used for evaluation due to activity limitations imposed by COVID-19.

**Figure:**
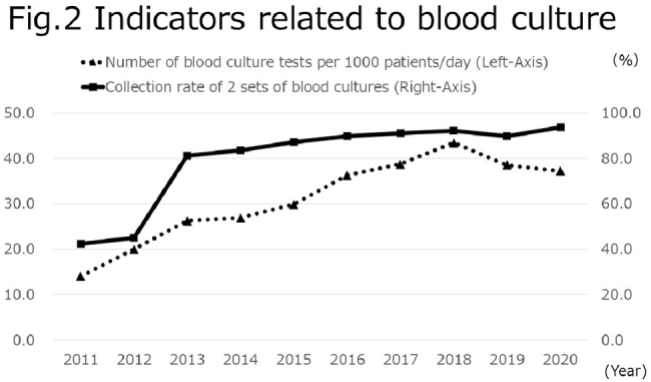

**Results:**

In terms of indicators related to MRSA, the nosocomial incidence of MRSA infections was 3.4‰ in 2011, but gradually decreased to 1.1‰ in 2020. The MRSA/*S. aureus* detection rate also declined from 47.0% to 33.4% during the same period (Fig. 1). On the other hand, the number of blood culture tests per 1000 patients/day decreased from 14.1 in 2011 to 37.3 in 2020, and the collection rate of 2 sets of blood cultures improved from 42.5% to 93.7% during the same period (Fig. 2).

**Conclusion:**

Bundling of various initiatives is important to improve the quality of hospital infection control. Although the Link Staff Program was not the only factor that improved quality, it is believed that this initiative was successful in providing knowledge and increasing awareness of infection control to all staff in all professions.

**Disclosures:**

**All Authors**: No reported disclosures

